# Lupus nephritis progression in FcγRIIB^-/-^yaa mice is associated with early development of glomerular electron dense deposits and loss of renal DNase I in severe disease

**DOI:** 10.1371/journal.pone.0188863

**Published:** 2017-11-30

**Authors:** Kjersti Daae Horvei, Hege Lynum Pedersen, Silje Fismen, Dhivya Thiyagarajan, Andrea Schneider, Ole Petter Rekvig, Thomas H. Winkler, Natalya Seredkina

**Affiliations:** 1 RNA and Molecular Pathology Research Group, Department of Medical Biology, Faculty of Health Sciences, UIT–The Arctic University of Norway, Tromsø, Norway; 2 Department of Biology, Nikolaus-Fiebiger-Zentrum für Molekulare Medizin, Friedrich-Alexander Universität Erlangen-Nürnberg, Erlangen, Germany; Instituto Nacional de Ciencias Medicas y Nutricion Salvador Zubiran, MEXICO

## Abstract

FcγRIIB^-/-^yaa mice develop severe lupus glomerulonephritis due to lack of an inhibitory immune cell receptor combined with a Y-chromosome linked autoimmune accelerator mutation. In the present study, we have investigated nephritis development and progression in FcγRIIB^-/-^yaa mice to find shared features with NZB/NZW F1 lupus prone mice and human disease. We sacrificed 25 male FcγRIIB^-/-^yaa mice at various disease stages, and grouped them according to activity and chronicity indices for lupus nephritis. Glomerular morphology and localization of electron dense deposits containing IgG were further determined by immune electron microscopy. Renal DNase I and pro-inflammatory cytokine mRNA levels were measured by real-time quantitative PCR. DNase I protein levels was assessed by immunohistochemistry and zymography. Our results demonstrate early development of electron dense deposits containing IgG in FcγRIIB^-/-^yaa mice, before detectable levels of serum anti-dsDNA antibodies. Similar to NZB/NZW F1, electron dense deposits in FcγRIIB^-/-^yaa progressed from being confined to the mesangium in the early stage of lupus nephritis to be present also in capillary glomerular basement membranes. In the advanced stage of lupus nephritis, renal DNase I was lost on both transcriptional and protein levels, which has previously been shown in NZB/NZW F1 mice and in human disease. Although lupus nephritis appears on different genetic backgrounds, our findings suggest similar processes when comparing different murine models and human lupus nephritis.

## Introduction

Lupus nephritis is a serious organ manifestation affecting 20–50% of patients with systemic lupus erythematosus (SLE) [1, 2]. Up to 25% of patients with lupus nephritis develop end stage renal disease despite the emergence of new immunomodulatory agents the last decades [3, 4]. The complex and partly unknown pathogenesis of SLE and lupus nephritis is challenging for development of new and specific treatments. A diversity of genetic variants has been identified to increase susceptibility to SLE and lupus nephritis, often in an epistatic manner [5]. Ethical and practical factors limit human studies, and murine models are therefore essential to identify the pathological impact of various genes. Common disease features in different murine models, independent of genetic background, often apply to human lupus nephritis, to become potential therapeutic targets [6].

The lupus-prone murine model FcγRIIB^-/-^yaa on a C57BL6 (B6)-background was generated and first described in 2002 by Bolland et al. In these mice, lack of inhibitory FcγRIIB on immune cells combined with the Y-chromosome linked autoimmune accelerator (*yaa*) mutation causes an especially severe variant of glomerulonephritis in male mice [7].

FcγRIIB is the only inhibitory member of Fcγ-receptors, which target IgG immune complexes [8, 9]. When normally expressed on professional antigen presenting cells, plasma cells, and granulocytes, FcγRIIB prevents autoimmunity by two main mechanisms. First, receptor stimulation on B-cells increases the threshold of B-cell activation, thereby decreasing the amount of autoantibodies produced. Second, receptor stimulation on professional antigen presenting cells at the site of inflammation suppresses antigen internalization and production of pro-inflammatory cytokines [10–12]. Decreased expression levels of FcγRIIB are associated with autoimmunity in humans [13–17] and in several lupus-prone murine models such as NZB, BXSB, MRL and NOD [18].

The *yaa*-mutation is an unbalanced translocation of at least 16 genes from the telomeric end of the X-chromosome onto the Y-chromosome [19, 20]. Among these genes, *toll like receptor 7 (TLR7)* is considered the most important gene for the *yaa* autoimmune phenotype [21, 22]. Activation of TLR7 leads to transcription of type I interferons [23] and the pro-inflammatory cytokines IL-6, IL-10 and TNFα through the NF-κB pathway [24–26], promoting inflammatory progression in lupus nephritis.

In FcγRIIB^-/-^yaa, an accelerating autoimmune inflammation is hence caused by increased exposure of endogenous nucleic antigens to intracellular TLRs, and especially excessive TLR7 signaling. Early development of anti-nuclear autoimmunity, splenomegaly and lethal glomerulonephritis are characteristic features of this model [7, 27–29]. Studies so far have implicated anti-dsDNA and anti-ribonuclear protein (anti-RNP) as the main anti-nuclear antibodies [27, 28].

In NZB/NZW F1, we have previously found a two-stepped disease development. The mice developed mesangial IgG-deposits along with anti-dsDNA antibodies in an early stage, and IgG-deposits in the glomerular basement membrane (GBM) with severe proteinuria in the end stage disease. The IgG-deposits in mesangium and GBM colocalized with DNA within electron dense structures (EDS). At the same time as development of end stage lupus nephritis, we observed downregulation of renal DNase I expression [30]. A similar pattern was also observed in human lupus nephritis. Glomerular IgG-deposits in EDS colocalized with DNA, and renal DNase I downregulation was limited to patients with EDS in the GBM [31]. In addition, renal gene expression profiling has showed downregulation of DNase I in nephritic NZM2410 and (NZWxBXSB) F1 mice [32].

As DNase I is the major renal endonuclease [33], loss of DNase I is likely to negatively affect local chromatin clearance. Based on the two-stepped disease model, we have earlier proposed that loss of renal DNase I lead to accumulation and exposure of chromatin along renal matrices and membranes, where it serves as a target for anti-chromatin antibodies. Binding of chromatin fragment-anti-IgG immune complexes to glomerular membranes may promote progression of mild mesangial lupus nephritis to end stage kidney disease [30].

Thus, the aim of the present study was to investigate if the two-stepped lupus nephritis development and loss of DNase I also applied for FcγRIIB^-/-^yaa mice. A special focus has been on histopathology, glomerular targets of anti-dsDNA antibodies, and expression levels of cytokines, toll like receptors (TLRs) and DNase I during disease progression. If nephritis in FcγRIIB^-/-^yaa resembles other lupus-prone murine models and the human form of the disease, this may guide us to new causal therapy strategies–irrespective of the genetic background.

## Material and methods

### Ethics statements

The study was approved by the Regional Animal Research Authority at the University of Erlangen-Nurnberg, Germany (Regierung von Mittelfranken, 54–2532.2-3/08 and TS-05/5).

### Mice

FcγRIIB^-/-^ mice[34] backcrossed for 12 generations to C57BI/6 were obtained from Taconic Biosciences and crossed with B6.yaa mice (obtained from Jackson Laboratories). 25 male FcγRIIB^-/-^ yaa mice were sacrificed at various disease stages by CO_2_ asphyxiation followed by cervical dislocation. Seven mice were aged 4–9 weeks and the remaining 18 mice were 26 weeks or older. Proteinuria and serum anti-dsDNA antibody titers were checked every month from 3–5 months of age. 24-hour urine collection was done immediately before the mice were sacrificed, and urine samples were stored at -80°. Four of the youngest mice did not have sera nor urine for evaluation. Isolated renal tissue from all 25 mice was snap frozen, preserved in 8% formalin for morphologic studies, and embedded in OCT for immunofluorescence analyses.

### Determination of proteinuria and serum antibodies

Urinary protein concentration was determined using protein quantification assay from Macherey-Nagel (M740967, Duren, Germany) according to the manufacturer instruction. Briefly urine was diluted to 1:10, mixed with Protein Solving Buffer and subsequently incubated for 30 minutes with Quantification Reagent. After incubation, light extinction is measured photometrically. The protein concentration is determined in reference to a BSA (Bovine Serum Albumin) calibration curve.

Anti-dsDNA antibodies in serum were detected using standard indirect ELISA. 96-well plates were coated with calf thymus DNA (Sigma) and incubated overnight. Wells were incubated with serial dilutions of sera in PBS/0.02% Tween for 1 hour, washed and incubated with anti-IgG antibodies (Sigma anti-mouse IgG A3673) for 1 hour. O-phenylenediamine dihydrochloride (Sigma P8787) in phosphate-citric buffer and 30% hydrogen peroxide were used as substrate, and the reaction was stopped using HCl. Optical density was read at 492 nm (Multiskan Ascent, Thermo Electron Corporation). Antibody titers were determined as the dilution giving 40% of maximum binding of a positive reference serum. PBS/0.05% Tween was used for washing between steps, and incubations were done at 37°C.

Anti-RNP antibodies in serum were detected using an ELISA kit according to the instruction manual (Alpha Diagnostic Intl. 5410).

### Immune electron microscopy (IEM) and colocalization IEM

For immune electron microscopy, renal samples were fixated in 8% formaldehyde in PBS and cortex samples were further processed in sucrose and glycine as described [35]. Ultrathin cryosections were incubated with goat anti-mouse IgG conjugated with 6-nm gold particles (Jackson ImmunoResearch Laboratories, Inc.) to correlate the morphological changes with the presence of antibodies bound in the glomeruli in vivo as described in [36]. The grids were contrasted with uranyl acetate and examined at x10-x40 K magnification using a JEM-1010 transmission electron microscope (Jeol, Tokyo, Japan). One glomerulus from each mouse was evaluated. Two mice were not evaluated because of insufficient quality of the sections. Presence of 1) mesangial EDS and 2) EDS in the capillary GBM were scored in a semi-quantitative way: 0, negative; 1+, 1–3 EDS locations; 2+, >3 EDS locations. In addition, podocyte foot process effacement was scored semi-quantitatively: 0, negative; 1+, average width of podocyte foot processes >1 μm; 2+, absence of podocyte foot processes.

To determine whether in vivo-bound autoantibodies were colocalized with extracellular chromatin, ultrathin cryosections were processed as described above. Sections were incubated with rabbit anti-mouse IgG (Cappel, ICN Pharmaceuticals, Inc.) and protein A conjugated with 5-nm gold particles (PAG-5nm, University of Urecht, The Netherlands), in addition to anti-dsDNA mAb DNA6 and protein A conjugated with 10-nm gold particles (PAG-10nm). Micrographs were taken using JEM-1010 transmission electron microscope (Jeol, Tokyo, Japan). The method is described in details in [36].

### Histology and immunohistochemistry (IHC)

Paraffin-embedded formalin fixated renal tissue were cut in 4 μm sections. For morphologic evaluation, sections with hematoxylin and eosin stain and periodic acid-schiff (PAS) stain were scored blinded by a pathologist for determination of activity index (0–24) and chronicity index (0–12), as formulated by Austin et al [37].

Protein expression of DNase I and TLR7 in renal sections were evaluated by immunohistochemistry. After deparaffinization and rehydration of 4 μm renal paraffin-embedded sections, sections were heated up to 97°C for 20 minutes in a citrate buffer pH 6.0. The sections were then blocked for 8 minutes with Peroxidase Block supplied by the kit, and 30 minutes for nonspecific hydrophobic binding with 10% goat serum (Invitrogen) in PBS. The sections were incubated with the primary antibody for 30 minutes (LifeSpan DNase I LS-B4846, Abcam TLR7 ab45371). Secondary antibody and chromogen-solution were applied according to the manufacturers manual (Dako EnVision+ HRP Rabbit (DAB+) kit, K4011). Counterstaining was then preformed using hematoxylin and Scotts solution.

### Direct immunofluorescence (IF) microscopy

IgG deposits in renal tissue were detected on OCT-embedded 4 μm cryosections. The sections were fixated in Hepes-buffered paraformaldehyde, and then blocked in 5% goat serum (Invitrogen) for 20 minutes. Next, the sections were incubated with F(ab’)-goat anti-mouse IgG (H+L) (Invitrogen, A-11018) and counterstained with DAPI (Abbot, 06J49-001).

### RNA isolation and cDNA synthesis

Total RNA from snap frozen kidneys was isolated by Quiagen miRNeasy Mini Kit (217004) according to their protocol. The RNA concentration was measured spectrophotometrically with NanoDrop (NanoDrop technologies). Samples were reverse transcribed using High Capacity cDNA Reverse Transcription Kit (ThermoFisher Scientific).

### Real-time quantitative PCR analysis

Real-time quantitative PCR was preformed using predesigned TaqMan Gene Expression Assays (ThermoFisher Scientific). TATA-box Bindi006Eg Protein (TBP) and β-actin were used as housekeeping gene controls. The following accession numbers for individual primers and probes were: IL-1β Mm9999061_m1, IL-6 Mm00446190_m1, IL-10 Mm9999062_m1, TNFα Mm00443258, IFNγ Mm01168134, TLR7 Mm00446590_m1, TLR8 Mm01157262_m1, TLR9 Mm00446193_m1, Eif2ak2 Mm01235643_m1, Ifit1 Mm00515153_m1, Mx1 Mm00487796_m1, DNase1 Mm01342389_g1, TBP Mm00446973_m1 and β-actin 4352933E. Relative expression levels of mRNA were calculated using the ΔΔCT method.

### Renal protein isolation

Protein isolation was done by homogenizing a cross-section of snap frozen renal tissue in DNase I Reaction Buffer with 0.1% Triton and 0.1% Protease Inhibitor Cocktail (P8340 Sigma), using Precellys24 (Bertin Instruments) 6000 rpm 2 x 15 seconds. Samples were centrifuged at 13.000 G for 5 min at 4°C. Protein concentration was measured using DirectDetect Infrared Spectrometer (Merk Millipore). Samples were normalized to protein concentration of 1.0 mg/ml, and used for single radial enzyme diffusion (SRED) and zymography analysis.

### Single radial enzyme diffusion (SRED)

DNase I activity was quantified using SRED. 1% agarose gel made on DNase I reaction buffer (40mM Tris, ph 7.6 / 2mM CaCl_2_ / 2mM MgCl_2_) was heated up until the solution was fully dissolved, and cooled down to 60°C before adding 300 μl salmon sperm DNA (Invitrogen) and 20 μl GelRed (Biotium, 41003) to a 100 ml solution. After the gel solidified, 3.5 μl of sample were loaded into wells of 1.0 mm diameter. An UV-picture was captured after 22 hours incubation at 37°C in a humid chamber, and the circle perimeter reflecting DNase I activity was measured. Recombinant human DNase I (DNASE15-R-100, Alpha Diagnostic Intl. Inc.) was used in four concentrations (0.1 IU, 0.025 IU, 0.0063 IU and 0.0016 IU) to make the standard curve for each gel.

### DNase I zymography

DNase I activity in the kidneys was detected by DNase I zymography, as described [38, 39]. Briefly, proteins were separated in 10% SDS-polyacrylamide gels containing 100 ng⁄ml heat-denatured salmon sperm DNA (Invitrogen Corp.). SDS was removed after electrophoresis by washing the gel in 2% Triton X-100 in miliQ water for 1 hour at room temperature. The gel was incubated in DNase I reaction buffer at 37°C for 18 hours and stained with GelRed for 30 minutes. UV-picture was captured to show dark areas were active DNase I had digested DNA in the gel. Recombinant human DNase I (DNASE15-R-100, Alpha Diagnostic Intl. Inc.) was used as a control.

### Statistics

Values in histograms are presented as mean with standard deviation. Differences between groups were analyzed with one-way ANOVA using a Games-Howell post hoc test. Spearman’s rank-order correlation coefficient (*r*_*s*_) was used for bivariate correlation analysis, with a two-tailed statistical significance. IBM SPSS statistics 24 software was used for statistical analysis. P-values <0.05 were considered significant.

## Results

### FcγRIIB^-/-^yaa mice develop glomerular EDS containing IgG in early disease stages, before positive anti-dsDNA titers and considerable proteinuria

**Fig 1:**
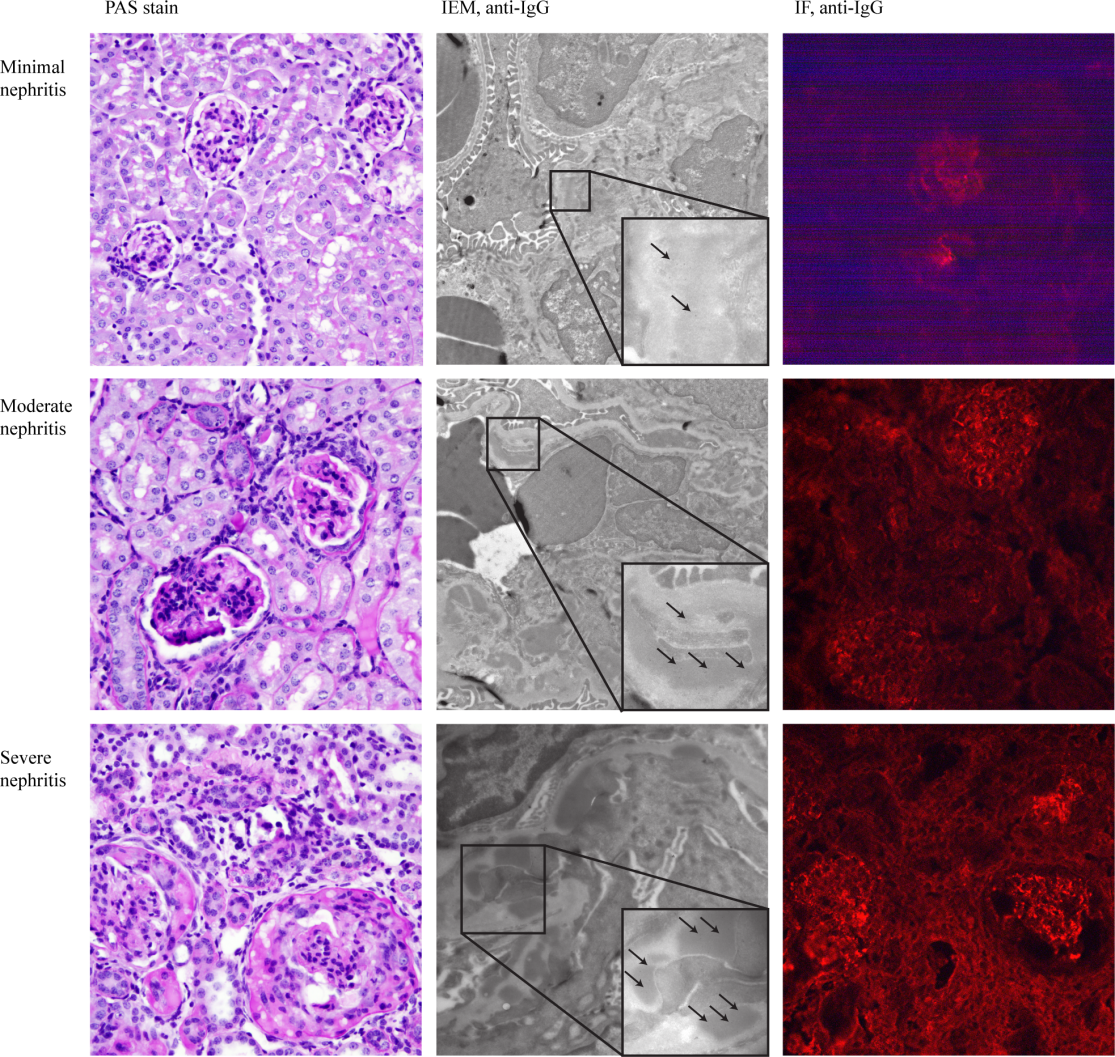
Renal morphology in different disease stages. Typical micrographs from minimal, moderate, and severe nephritis. Periodic acid-Schiff (PAS) staining in the left panel show increasing changes in glomeruli and tubulointerstitium with disease progression: endocapillary proliferation, infiltration of immune cells, formation of wire loops and hyaline thrombi, crescents, atrophy and sclerosis. The middle panel demonstrate immune electron microscopy (IEM) with anti-IgG antibodies (6 nm gold) showing increasing amounts of IgG-deposits (arrows) in mesangium and later also in the capillary glomerular basement membrane. In addition, podocyte effacement was apparent in moderate and severe nephritis. Immunofluorescence (IF) in the right panel shows IgG staining in glomeruli for all three groups, and also staining in the tubulointerstitium in mice with moderate and severe nephritis.

**Fig 2:**
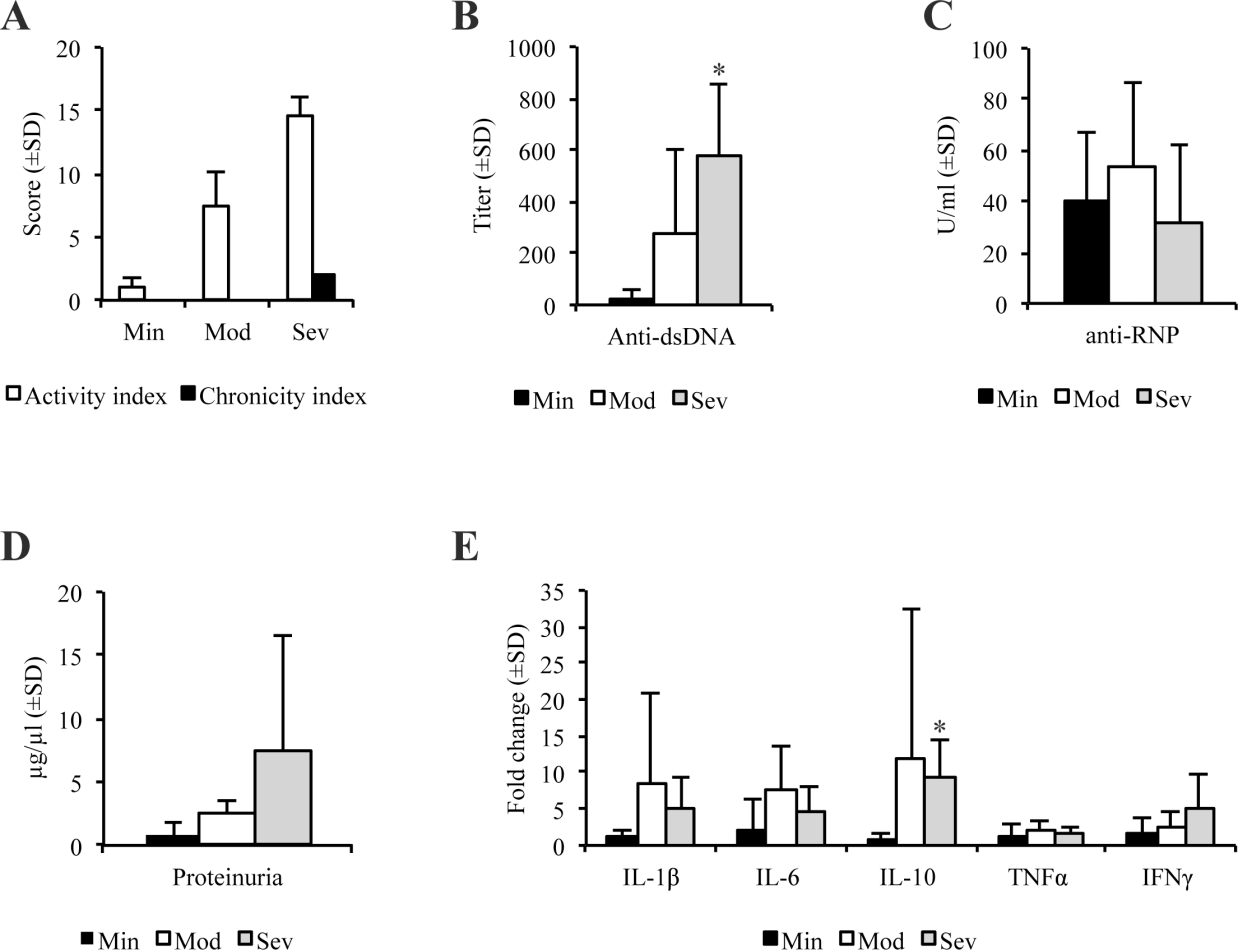
General disease features of lupus nephritis. Mice with minimal nephritis (Min, n = 9), moderate nephritis (Mod, n = 11) and severe nephritis (Sev, n = 5) were compared according to different disease parameters. 20/25 mice had end-stage sera and urine available for ELISA and proteinuria evaluation: Min n = 5, Mod n = 10, Sev n = 5. (A) Distribution of activity and chronicity indices of renal tissue in the different groups, presented as mean with standard deviations. Mean activity index was highest in the severe nephritis group, which also had chronic changes. (B) Mean anti-dsDNA antibody titer with standard deviations. Mice with severe nephritis had elevated anti-dsDNA antibody titer compared to minimal nephritis (p = 0.023). (C) Mean anti-RNP activity concentrations (U/ml) with standard deviations. (D) Urinary protein concentrations (μg/μl) with standard deviations. (E) Renal cytokine mRNA levels, presented as fold change relative to the mice with minimal nephritis. IL-10 was significantly upregulated in mice with severe nephritis compared to minimal nephritis (p = 0.041).

General disease features of lupus nephritis in FcγRIIB^-/-^yaa mice were evaluated by renal microscopy (Fig 1, Fig 2A), presence of serum anti-dsDNA and anti-RNP antibodies (Fig 2B and 2C), proteinuria (Fig 2D) and renal expression levels of pro-inflammatory cytokines (Fig 2E). Mice were grouped according to activity and chronicity indices for lupus nephritis: minimal nephritis (activity index ≤2, chronicity index 0), moderate nephritis (activity index >2, chronicity index 0) and severe nephritis (chronicity index ≥1) (S1 Table). Mice with severe nephritis also had the highest activity index (Fig 2A).

In the minimal nephritis group, seven of nine animals were 4–9 weeks old (Table 1), and had changes in renal morphology such as glomerular endocapillary proliferation, glomerular neutrophil infiltration and/or presence of mononuclear cells in the interstitium (Fig 1, PAS staining; S1 Table). 66% of mice with minimal nephritis had various degrees of mesangial EDS containing IgG, and 22% had additional EDS containing IgG in the capillary GBM (Table 1). Immunofluorescence showed some but weak IgG staining in glomeruli (Fig 1). All mice in this group had normal morphology of podocyte foot processes, and no or negligible proteinuria (Table 1; Fig 2D). Most of them were negative for anti-dsDNA antibodies, and the only mouse with a very low positive titer was older than 26 weeks (Fig 2B; Table 1; S1 Fig). However, all mice with minimal nephritis had detectable levels of anti-RNP antibodies (Fig 2C).

**Table 1:** Distribution of various lupus nephritis parameters within different disease stages.

	Age	EDS mesangium*		EDS capillary GBM*		Podocyte effacement*		Anti-dsDNA antibodies^a^		Proteinuria/ μg/μl^b^	
**Minimal nephritis**	≤9 weeks: 7 (78%)	Score 0:	3 (33%)	Score 0:	7 (78%)	Score 0:	9 (100%)	Neg:	4 (80%)	<0.5:	3 (60%)
		Score 1:	3 (33%)	Score 1:	2 (22%)	Score 1:	-	Pos:	1 (20%)	0.5–5:	2 (40%)
	≥26 weeks: 2 (22%)	Score 2:	3 (33%)	Score 2:	-	Score 2:	-			>5:	-
**Moderate nephritis**	≤9 weeks: -	Score 0:	-	Score 0:	2 (20%)	Score 0:	4 (40%)	Neg:	4 (40%)	<0.5:	-
	≥26 weeks: 11 (100%)	Score 1:	-	Score 1:	6 (60%)	Score 1:	5 (50%)	Pos:	6 (60%)	0.5–5:	8 (80%)
		Score 2:	10 (100%)	Score 2:	2 (20%)	Score 2:	1 (10%)			>5:	2 (20%)
**Severe nephritis**	≤9 weeks: -	Score 0:	-	Score 0:	-	Score 0:	-	Neg:	-	<0.5:	-
	≥26 weeks: 5 (100%)	Score 1:	-	Score 1:	1 (25%)	Score 1:	-	Pos:	5 (100%)	0.5–5:	2 (40%)
		Score 2:	4 (100%)	Score 2:	3 (75%)	Score 2:	4 (100%)			>5:	3 (60%)

Mice were divided into groups according to renal histological changes scored by activity and chronicity indices for lupus nephritis: minimal nephritis, n = 9; moderate nephritis, n = 11; and severe nephritis, n = 5. The different lupus nephritis parameters were: age, the degree of electron dense structures (EDS) in mesangium (score 0–2), the degree of EDS in the glomerular basement membrane (GBM) lining capillary lumen (score 0–2), the degree of podocyte foot process (PFP) effacement (0: normal morphology, 1: fusion of PFP, 2: loss of PFP), the presence of anti-dsDNA antibodies, and the level of proteinuria.

*23/25 mice had sufficient quality of sections for IEM evaluation.

^a^20/25 mice had end-point sera available for anti-dsDNA ELISA.

^b^20/25 mice had end-point urine available for proteinuria evaluation.

11 mice were included in the moderate nephritis group, and were all 26 weeks or older (Table 1). Characteristic histopathologic features of these mice were endocapillary proliferation and enlargement of glomeruli, infiltration of inflammatory cells in glomeruli and interstitium, and formation of wire loops and hyaline thrombi. Some mice also developed cellular crescents and glomerular fibrinoid necrosis (Fig 1, PAS staining; S1 Table). IEM revealed massive mesangial EDS containing IgG in all mice with moderate nephritis, while 80% had some degree of EDS containing IgG localized in the subendothelial and/or the subepithelial area of capillary GBM (Table 1). IgG deposits in mesangium and along capillary walls were also demonstrated by immunofluorescence (Fig 1). Podocyte effacement was apparent in 60% of animals. Most mice had a positive anti-dsDNA titer and proteinuria (Fig 2B and 2D; Table 1; S1 Fig). Serum anti-RNP antibodies were detected in all mice with moderate nephritis. However, anti-RNP activity concentrations were similar to those detected in mice with minimal nephritis (Fig 2C).

Five mice had severe nephritis, all 26 weeks or older (Table 1). In addition to pathologic changes associated with active nephritis, these mice also presented initial chronic changes with fibrous crescents and tubular atrophy (Fig 1, PAS staining; S1 Table). All mice had EDS containing IgG in the mesangium and in the capillary GBM, and podocyte effacement by IEM (Table 1). As for the moderate nephritis group, anti-IgG immunofluorescence reflected the heavy burden of IgG-deposits seen by IEM (Fig 1). All mice had positive anti-dsDNA titers and proteinuria (Fig 2B and 2D; Table 1, S1 Fig). Serum anti-RNP activity concentrations remained the same as detected in mice with minimal and moderate nephritis (Fig 2C).

### Renal proinflammatory cytokines are elevated in moderate and severe disease in FcγRIIB^-/-^yaa mice

Renal mRNA expression levels of IL-1β, IL-6, IL-10, TNFα and IFNγ were analyzed to evaluate how the local inflammatory milieu correlated to morphologic changes. Relative expression levels in mice with moderate and severe nephritis were calculated using the average expression level of minimal nephritis group as a reference. As demonstrated in Fig 2E, the renal proinflammatory cytokines were in general elevated in moderate and severe nephritis, whereas IL-10 was significantly upregulated in severe nephritis.

### Colocalization IEM revealed in vivo bound IgG and DNA in glomerular EDS in FcγRIIB^-/-^yaa mice

**Fig 3:**
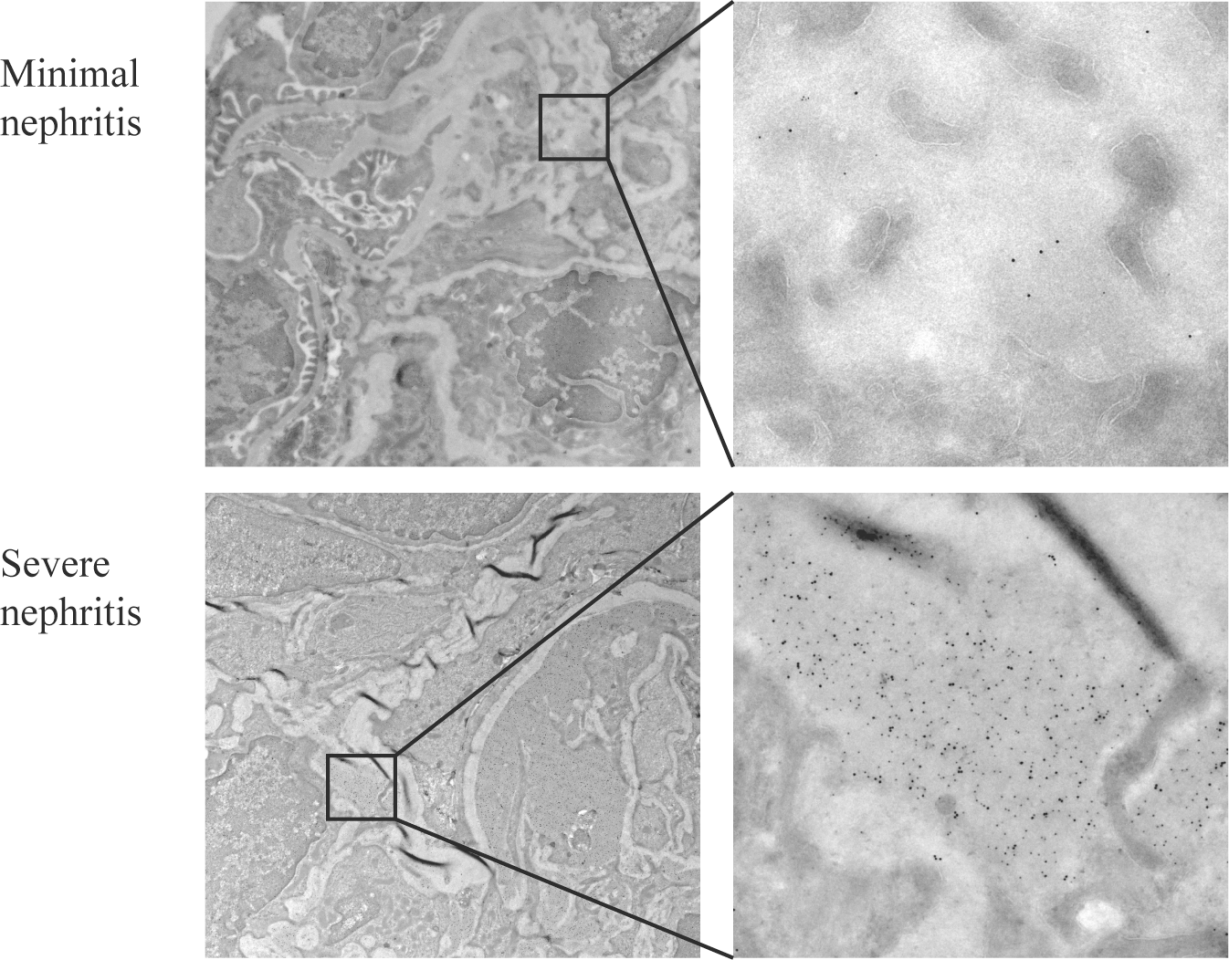
IgG and DNA colocalization immune electron microscopy. Renal sections from mice with minimal nephritis (upper panel) and severe nephritis (lower panel) were incubated with anti-IgG (5 nm gold) and anti-DNA (10 nm gold). IgG were restricted to EDS, and colocalized with DNA. Both IgG and DNA appeared with highest density in severe nephritis.

Renal sections with minimal nephritis and severe nephritis were analyzed by colocalization IEM to characterize the glomerular targets of anti-ds DNA antibodies. As demonstrated in Fig 3, IgG-bound 5 nm gold particles colocalized with DNA-bound 10 nm gold particles almost exclusively within EDS. IgG and DNA colocalization was confirmed in EDS of both minimal nephritis and severe nephritis. The density of both 5 nm and 10 nm gold particles was much higher in the late stage of the disease.

### In FcγRIIB^-/-^yaa mice, the activity index has highly significant correlations with IEM-evaluated glomerular disease features, proteinuria and anti-dsDNA antibody titer

We used correlation analysis to identify the strongest factors having impact on lupus nephritis progression in FcγRIIB^-/-^yaa. As shown in Table 2, we used Spearman’s correlation on multiple variables such as age, renal morphological changes, proteinuria, and presence of anti-dsDNA antibodies. The activity index correlated significantly with age (p = 0.002), mesangial EDS containing IgG (p = 0.001), EDS containing IgG in capillary GBM (p = 0.001), podocyte effacement (p<0.001), proteinuria (p = 0.037), and anti-dsDNA antibody titer (p = 0.002). Proteinuria and anti-dsDNA antibodies also showed significant correlations with most parameters. Age on the other hand correlated only significantly with the amount of mesangial EDS (p<0,001).

**Table 2:** Correlations between different variables in FcγRIIB^-/-^.yaa mice.

		Age	Activity index	EDS mesangium	EDS capillary GBM	Podocyte effacement	Proteinuria	Anti-dsDNA titer	DNase I mRNA	TLR7 mRNA
**Age**	*r*_s_*	1	0.591	0.708	0.411	0.329	0.136	0.431	-0.062	0.608
	p value^a^		0.002	0.000	0.051	0.125	0.566	0.058	0.770	0.001
	n^b^	25	25	23	23	23	20	20	25	25
**Activity index**	*r*_s_*	0.591	1	0.653	0.639	0.808	0.469	0.643	-0.585	0.798
	p value^a^	0.002		0.001	0.001	0.000	0.037	0.002	0.002	0.000
	n^b^	25	25	23	23	23	20	20	25	25
**EDS mesangium**	*r*_s_*	0.708	0.653	1	0.540	0.495	0.491	0.357	-0.086	0.624
	p value^a^	0.000	0.001		0.008	0.016	0.039	0.146	0.696	0.001
	n^b^	23	23	23	23	23	18	18	23	23
**EDS capillary GBM**	*r*_s_*	0.411	0.639	0.540	1	0.599	0.453	0.580	-0.490	0.595
	p value^a^	0.051	0.001	0.008		0.003	0.059	0.012	0.018	0.003
	n^b^	23	23	23	23	23	18	18	23	23
**Podocyte effacement**	*r*_s_*	0.329	0.808	0.495	0.599	1	0.609	0.438	-0.547	0.605
	p value^a^	0.125	0.000	0.016	0.003		0.007	0.069	0.007	0.002
	n^b^	23	23	23	23	23	18	18	23	23
**Proteinuria**	*r*_s_*	0.136	0.469	0.491	0.453	0.609	1	0.503	-0.263	0459
	p value^a^	0.566	0.037	0.039	0.059	0.007		0.028	0.262	0.042
	n^b^	20	20	18	18	18	20	19	20	20
**Anti-dsDNA titer**	*r*_s_*	0.431	0.643	0.357	0.580	0.438	0.503	1	-0.566	0.671
	p value^a^	0.058	0.002	0.146	0.012	0.069	0.028		0.009	0.001
	n^b^	20	20	18	18	18	19	20	20	20
**DNase I mRNA**	*r*_s_*	-0.062	-0.585	-0.086	-0.490	-0.547	-0.263	-0.566	1	-0.468
	p value^a^	0.770	0.002	0.696	0.018	0.007	0.262	0.009		0.018
	n^b^	25	25	23	23	23	20	20	25	25
**TLR7 mRNA**	*r*_s_*	0.608	0.798	0.624	0.595	0.605	0459	0.671	-0.468	1
	p value^a^	0.001	0.000	0.001	0.003	0.002	0.042	0.001	0.018	
	n^b^	25	25	23	23	23	20	20	25	25

The relationship between age, activity index for lupus nephritis, amount of mesangial electron dense structures (EDS), amount of EDS in the glomerular basement membrane (GBM) lining capillary lumen, degree of podocyte effacement, proteinuria, anti-dsDNA antibody titer, and renal mRNA expression levels of DNase I and TLR7 were scored.

*Spearman’s correlation coefficient.

^a^Two-tailed statistical significance.

^b^Number of mice included in the correlation analysis.

### TLR7 is expressed in tubular epithelial cells in FcγRIIB^-/-^yaa mice

**Fig 4:**
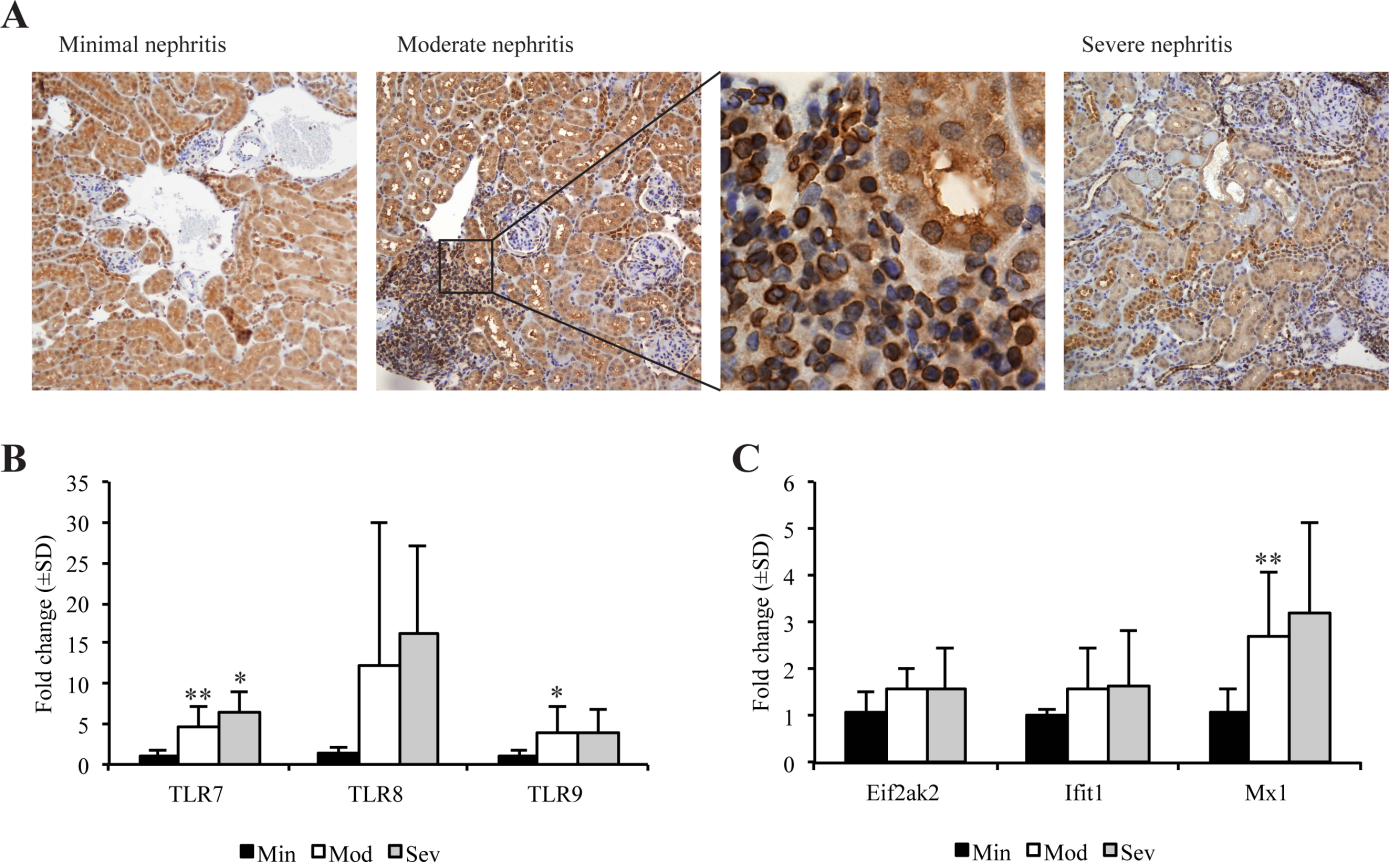
Renal expression of TLRs and interferon stimulated genes. Different disease stages were compared: Minimal nephritis (Min, n = 9), moderate nephritis (Mod, n = 11), and severe nephritis (Sev, n = 5). (A) IHC demonstrated renal TLR7 expression in tubular epithelial cells in mice with different stages of FcγRIIB^-/-^yaa nephritis. The perinuclear staining pattern of tubular epithelial cells resembled the staining pattern of infiltrating immune cells, as demonstrated for moderate nephritis (enlarged section). (B) Renal mRNA levels of TLR7, TLR8 and TLR9 from FcγRIIB^-/-^yaa mice with different stages of nephritis, presented as fold change relative to the minimal nephritis group. Expression levels of TLR7 and TLR9 were significantly upregulated in mice with moderate nephritis compared to mice with minimal nephritis (p = 0.006 and p = 0.027, respectively). In mice with severe nephritis, TLR7 was significantly upregulated compared to minimal nephritis (p = 0.017). (C) Renal mRNA expression levels of the interferon stimulated genes Eif2ak2, Ifit1 and Mx1 in FcγRIIB^-/-^yaa mice with different stages of nephritis, presented as fold change relative to the minimal nephritis group. Mx1 was significantly upregulated in moderate nephritis compared to mice with minimal nephritis (p = 0.008).

Since TLR7 is duplicated as a result of the *yaa*-mutation, we used IHC analysis to study the renal protein expression in different disease stages. Surprisingly, we found TLR7 staining in tubular epithelial cells in all groups of FcγRIIB^-/-^yaa mice, in addition to the expected staining of immune cells (Fig 4A). The staining pattern of tubular epithelial cells resembled the perinuclear staining pattern of immune cells in lymphoid aggregates in the kidneys (Fig 4A).

### Renal levels of TLRs and interferon stimulated genes are upregulated in moderate and severe lupus nephritis in FcγRIIB^-/-^yaa mice

We analyzed renal mRNA expression levels of TLR7, TLR8 and TLR9 in different disease stages to evaluate the potential of receptor activation and downstream transcription of type I interferons. As seen in Fig 3B, TLR7 was significantly upregulated in mice with moderate and severe nephritis, and TLR9 was significantly upregulated in moderate nephritis (Fig 3B). mRNA expression of TLR7 correlated well with mRNA expression of TLR8 (*r*_*s*_: 0.973, p<0.001) and TLR9 (*r*_*s*_: 0.812, p<0.001), as well as all disease parameters presented in Table 2.

To detect local interferon signaling, we investigated renal expression of the interferon-stimulated genes Eif2ak2, Ifit and Mx1 (Fig 4C). Mx1 was significantly upregulated in moderate nephritis.

### Renal DNase I is downregulated in severe lupus nephritis in FcγRIIB^-/-^yaa

**Fig 5:**
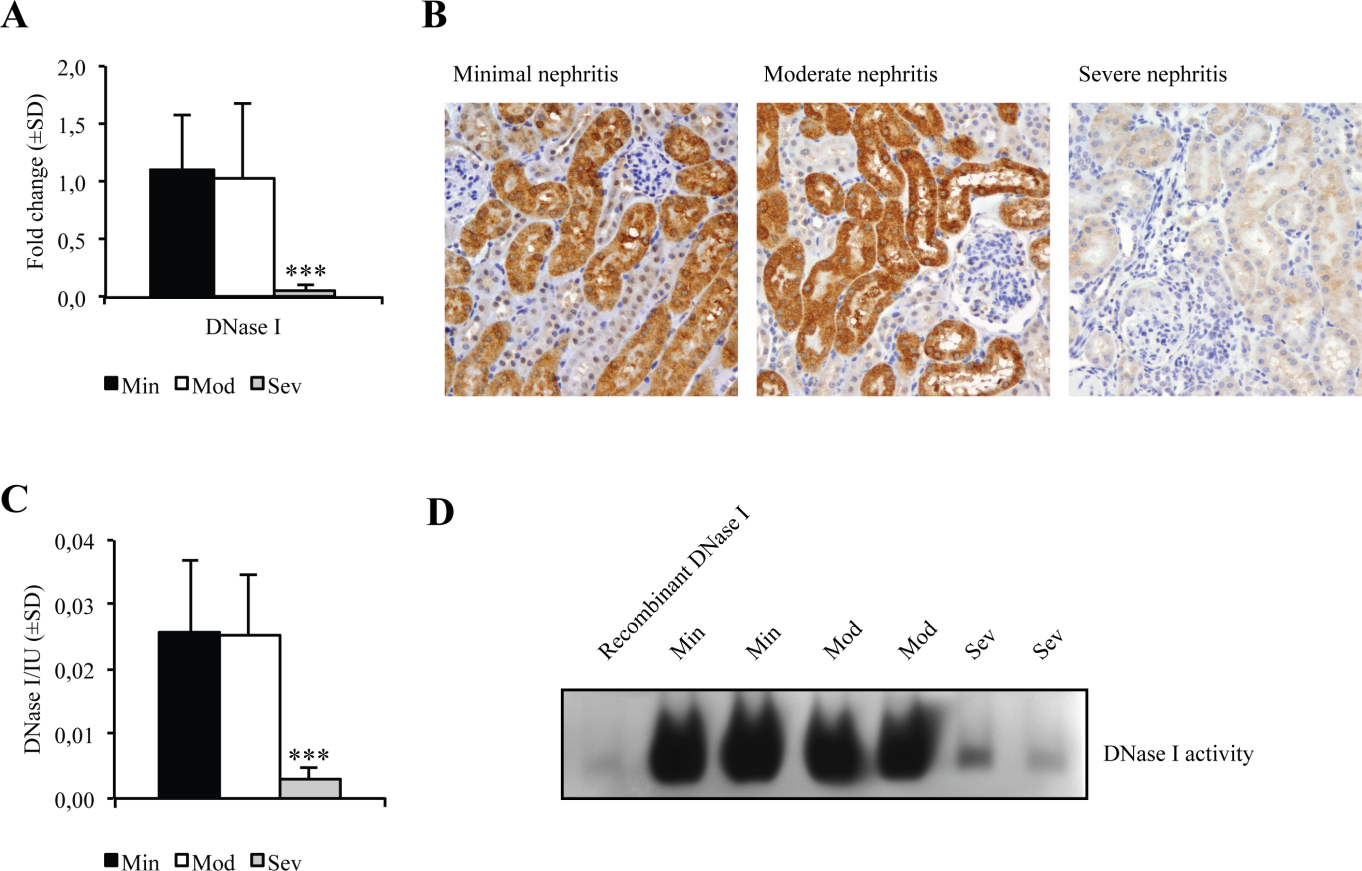
Renal DNase I expression. Different disease stages were compared: minimal nephritis (Min, n = 9), moderate nephritis (Mod, n = 11), and severe nephritis (Sev, n = 5). DNase I expression was downregulated in mice with severe nephritis on transcriptional, protein end enzyme activity levels. (A) Fold change of renal DNase I mRNA levels in mice with moderate and severe lupus nephritis relative to the minimal nephritis group, showing a highly significant downregulation in mice with severe nephritis compared to minimal nephritis and moderate nephritis (p<0.001 and p = 0.002, respectively). (B) Immunohistochemistry demonstrated tubular staining of DNase I in mice with minimal nephritis and moderate nephritis, and weak staining in mice with severe nephritis. (C) Renal DNase I enzyme activity measured by single radial enzyme diffusion. The enzyme activity was significantly lower in mice with severe nephritis compared to the minimal nephritis and moderate nephritis (p = 0.001 and p<0.001, respectively). (D) DNase I gel zymography showing enzyme activity of two typical samples from each group of mice. The enzyme activity was lower in mice with severe nephritis compared to the minimal and moderate nephritis group. Recombinant DNase I was used as a control (left band).

In order to verify whether progression of lupus nephritis in FcγRIIB^-/-^yaa mice was associated with loss of DNase I, we analyzed mRNA, protein levels and enzyme activity of renal DNase I in different disease stages. As demonstrated in Fig 5A, mRNA levels of DNase I were markedly reduced in kidneys with severe nephritis, while mice with moderate disease had similar mRNA expression levels to mice with minimal nephritis. The low DNase I mRNA levels in severe nephritis were also reflected by low renal protein expression, as demonstrated by IHC analysis (Fig 5B), and renal enzyme activity as demonstrated by single radial enzyme diffusion assay (Fig 5C) and DNase I gel zymography (Fig 5D).

Thus, late stage of lupus nephritis in FcγRIIB^-/-^yaa mice is characterized by loss of renal DNase I on the gene expression, protein expression and enzyme activity levels. As demonstrated in Table 2, there is a significant negative correlation between DNase I mRNA levels and activity index (p = 0.002), the amount of EDS containing IgG in capillary GBM (p = 0.018), podocyte effacement (p = 0.007), and anti-dsDNA antibody titer (p = 0.009).

## Discussion

FcγRIIB^-/-^yaa mice developed glomerular EDS containing IgG before early diagnostic signs such as measurable titers of anti-dsDNA antibodies and proteinuria. Young animals (4–9 weeks) presented primarily mesangial EDS, while older mice with increasing signs of active nephritis also developed EDS in the capillary GBM. The capillary GBM deposits appeared at the same time as positive anti-dsDNA titers, but before heavy proteinuria and loss of renal DNase I. In addition, TLR7 was expressed by tubular epithelial cells, and was highly upregulated in moderate and severe disease.

In contrast to human disease, there is no consensus on classification of murine lupus nephritis. Assuming a homogenous disease progression, age is commonly used as a classification criterion in lupus-prone murine models. However, in the present study and in our previous longitudinal study of NZB/NZW F1 mice [30], we found considerable age-variation in disease development. Similar to human classification, presence and localization of EDS were used to group NZB/NZW F1 mice, but was less suitable for FcγRIIB^-/-^yaa mice in this study because of early EDS development. Here, we adapted activity and chronicity indices used for human lupus nephritis to our murine study. As pointed out in our correlation analysis, renal activity score for lupus nephritis correlated well with common disease parameters such as glomerular EDS, proteinuria and anti-dsDNA antibodies. Activity and chronicity indices were therefore used as classification criteria for different disease stages in FcγRIIB^-/-^yaa mice.

In FcγRIIB^-/-^yaa, the negative anti-dsDNA antibody titer in the minimal nephritis group may indicate that immune complexes in EDS contain other antigens than DNA. We detected anti-RNP antibodies in all mice, including mice with minimal nephritis. Also previous studies on FcγRIIB^-/-^yaa have found elevated levels of anti-RNP antibodies in an earlier stage compared to anti-dsDNA and anti-chromatin antibodies [27, 28]. Similar to the NZB/NZW F1 study, we here demonstrate colocalization of IgG and DNA in early mesangial deposits as well as in later deposits in GBM by IEM, indicating that low serum levels of anti-dsDNA antibodies may be nephritogenic in very early disease. As serum levels of antibodies only reflect the amount of the unbound fraction, fully absorbed anti-dsDNA antibodies to chromatin in the kidney would result in negative to low serum titers.

As lupus nephritis progressed in FcγRIIB^-/-^yaa mice, mesangial EDS containing IgG were increasingly prominent, and appeared before additional EDS containing IgG in the capillary GBM. EDS in subendothelial and subepithelial areas of GBM appeared in moderate nephritis associated with positive anti-dsDNA titers and proteinuria. Lupus nephritis progression through a mesangial stage before GBM deposits is also thoroughly reported for NZB/NZW F1 mice [30]. In human lupus nephritis, two recent studies following class II patients described progression to class III or IV despite treatment [40, 41], although class II mesangial nephritis traditionally is considered benign without progression [42].

Loss of DNase I was apparent in severe lupus nephritis in FcγRIIB^-/-^yaa mice. Although loss of DNase I in late stage disease is well known in other models, the cause and impact of DNase I silencing is still unclear. The role of DNase I in lupus nephritis development has been discussed for the last 40 years. The idea of chromatin-containing immune complexes due to reduced serum DNase I levels suggested DNase I as a promising substitutive therapy [43]. However, intravenous and subcutaneous administration of recombinant DNase I in lupus nephritis patients and NZB/NZW F1 mice did not show any impact on kidney function or disease activity [44, 45]. These results may indicate that serum DNase I is not important in the degradation of dying renal cells, and that renal DNase I levels could be important for local chromatin degradation.

DNase I is mainly expressed in tubular epithelial cells, where it represent 80% of all endonuclease activity [33, 46]. However, DNase I is also found in glomeruli [31]. In NZB/NZW F1 mice, loss of renal DNase I occurred simultaneously with appearance of DNA-IgG complexes in GBM and severe proteinuria [30]. In FcγRIIB^-/-^yaa mice, capillary EDS in glomeruli appeared in early disease stages, before heavy proteinuria and loss of DNase I. Thus, in this study, loss of DNase I cannot explain the widespread glomerular IgG deposits in moderate nephritis. Recently, it was shown that anti-dsDNA antibodies bind to tubular epithelial cells, contributing to tubulointerstitial inflammation [47]. Tubular DNase I levels may therefore affect the amount of chromatin-containing immune complexes and the overall renal inflammation. Interestingly, FcγRIIB^-/-^yaa mice with initial chronic inflammatory changes in the kidney showed reduced DNase I expression. Chronic inflammatory signaling pathways may play an important role in promoting downregulation of DNase I.

TLR7 was highly upregulated in renal tissue during disease progression. TLR7 is normally only expressed in plasmacytoid dendritic cells, macrophages and B cells [48, 49]. In this study we also found TLR7 expressed by tubular epithelial cells in FcγRIIB^-/-^yaa, a possible outcome of the *TLR7* gene duplication. Upregulation of TLR7 expression in nephritic mice, as well as highly significant correlation with TLR9 expression levels, indicate that the disease process itself also contributes to TLR7 expression.

Tubular epithelial cells are well known for their capability to become activated by interferon gamma to express MHC class II and co-stimulatory molecule B7, and thereby function as antigen-presenting cells with the capacity to prime T cells [50, 51]. A recent study on human lupus nephritis found renal tubular epithelial cells to be the main producers of IFNα, at the same time as IFNα induced expression of TLR3 in the same cells [52]. Thus, expression of TLR7 in tubular cells, in addition to professional antigen presenting cells, may contribute to local interferon levels and transcription of other proinflammatory cytokines, driving lupus nephritis progression.

## Conclusions

FcγRIIB^-/-^yaa mice develop early morphologic changes characteristic of lupus nephritis. Before detectable levels of serum anti-dsDNA antibodies, we observed glomerular proliferation, immune cell infiltration, and development of glomerular EDS containing IgG in kidneys of FcγRIIB^-/-^yaa mice. These findings might reflect the excessive TLR7 signaling and subsequent upregulation of type I interferons and pro-inflammatory cytokines, causing a stronger autoimmune tendency in FcγRIIB^-/-^yaa compared to other lupus-prone murine models.

Despite of differences in genetic background causing autoimmunity, lupus nephritis progression in FcγRIIB^-/-^yaa mice shares characteristics with human disease and other lupus-prone murine models. Glomerular EDS progress from being confined to the mesangium in the early stage of lupus nephritis to be present also in GBM. In severe lupus nephritis, DNase I is lost on both transcriptional and protein levels. This study suggests chronic inflammatory pathways to cause DNase I downregulation, which may also apply to human disease and other lupus prone models.

## Supporting information

S1 Table Scoring of activity index and chronicity index in individual mice. Each component was scored 0–3, and “cellular crescents” and “fibrinoid necrosis or karyorrhexis” was in addition weighted by a factor of 2. Mice were grouped according to this scoring; Minimal nephritis (Min): activity index ≤2, chronicity index 0. Moderate nephritis (Mod): activity index >2, chronicity index 0. Severe nephritis (Sev): chronicity index ≥1.
(DOCX)

S1 Fig Anti-dsDNA antibody titers in individual mice. 6/25 mice had sera available for anti-dsDNA ELISA at several time points in addition to end stage sera.
(TIF)
