# Supplementary material for: Lupus nephritis progression in FcγRIIB-/-yaa mice is associated with early development of glomerular electron dense deposits and loss of renal DNase I in severe disease
Source: PLoS One. 2017 Nov 30;12(11):e0188863. doi: 10.1371/journal.pone.0188863 (PMC5708736; doi:10.1371/journal.pone.0188863)
Supplement: S1 Table — Each component was scored 0–3, and “cellular crescents” and “fibrinoid necrosis or karyorrhexis” was in addition weighted by a factor of 2. Mice were grouped according to this scoring; Minimal nephritis (Min): activity index ≤2, chronicity index 0. Moderate nephritis (Mod): activity index >2, chronicity index 0. Severe nephritis (Sev): chronicity index ≥1. (DOCX) [file pone.0188863.s001.docx]

**S1 Table. Scoring of activity index and chronicity index in individual mice.**

| **Mouse** | **Group** | Cellular crescents | Glomerular endocapillary hypercellularity | Leukocyte infiltration | Fibrinoid necrosis or karyorrhexis | Hyaline thromni,  wire loops | Mononuclear cells tubulointerstitium | **Activity index** | Glomerular sclerosis | Fibrous crescents | Interstitial fibrosis | Tubular atrophy | **Chronicity index** |
| --- | --- | --- | --- | --- | --- | --- | --- | --- | --- | --- | --- | --- | --- |
| 2356 | Min | 0 | 0 | 0 | 0 | 0 | 0 | **0** | 0 | 0 | 0 | 0 | **0** |
| 4869 | Min | 0 | 0 | 0 | 0 | 0 | 0 | **0** | 0 | 0 | 0 | 0 | **0** |
| Y1 | Min | 0 | 1 | 0 | 0 | 0 | 0 | **1** | 0 | 0 | 0 | 0 | **0** |
| 2134 | Min | 0 | 0 | 1 | 0 | 0 | 0 | **1** | 0 | 0 | 0 | 0 | **0** |
| 4887 | Min | 0 | 1 | 0 | 0 | 0 | 0 | **1** | 0 | 0 | 0 | 0 | **0** |
| 4730 | Min | 0 | 0 | 0 | 0 | 0 | 1 | **1** | 0 | 0 | 0 | 0 | **0** |
| 2135 | Min | 0 | 1 | 1 | 0 | 0 | 0 | **2** | 0 | 0 | 0 | 0 | **0** |
| 2449 | Min | 0 | 1 | 1 | 0 | 0 | 0 | **2** | 0 | 0 | 0 | 0 | **0** |
| C26 | Min | 0 | 1 | 1 | 0 | 0 | 0 | **2** | 0 | 0 | 0 | 0 | **0** |
| G23 | Mod | 0 | 2 | 1 | 0 | 1 | 0 | **4** | 0 | 0 | 0 | 0 | **0** |
| C32 | Mod | 0 | 1 | 1 | 0 | 0 | 2 | **4** | 0 | 0 | 0 | 0 | **0** |
| G28 | Mod | 0 | 2 | 1 | 0 | 1 | 1 | **5** | 0 | 0 | 0 | 0 | **0** |
| G26 | Mod | 0 | 2 | 2 | 0 | 2 | 1 | **7** | 0 | 0 | 0 | 0 | **0** |
| G29 | Mod | 0 | 3 | 2 | 0 | 1 | 1 | **7** | 0 | 0 | 0 | 0 | **0** |
| G17 | Mod | 0 | 3 | 2 | 0 | 2 | 1 | **8** | 0 | 0 | 0 | 0 | **0** |
| C9 | Mod | 0 | 2 | 2 | 0 | 2 | 2 | **8** | 0 | 0 | 0 | 0 | **0** |
| C10 | Mod | 0 | 2 | 2 | 0 | 2 | 2 | **8** | 0 | 0 | 0 | 0 | **0** |
| C27 | Mod | 2 | 2 | 2 | 0 | 2 | 1 | **9** | 0 | 0 | 0 | 0 | **0** |
| G24 | Mod | 2 | 2 | 1 | 2 | 2 | 2 | **11** | 0 | 0 | 0 | 0 | **0** |
| G21 | Mod | 2 | 2 | 2 | 2 | 2 | 2 | **12** | 0 | 0 | 0 | 0 | **0** |
| C23 | Sev | 2 | 2 | 1 | 2 | 3 | 2 | **12** | 0 | 1 | 0 | 1 | **2** |
| Y9 | Sev | 4 | 2 | 1 | 2 | 2 | 3 | **14** | 0 | 1 | 0 | 1 | **2** |
| G13 | Sev | 4 | 2 | 2 | 2 | 3 | 1 | **14** | 0 | 1 | 0 | 1 | **2** |
| G16 | Sev | 4 | 2 | 2 | 2 | 3 | 1 | **14** | 0 | 1 | 0 | 1 | **2** |
| G25 | Sev | 4 | 2 | 2 | 4 | 2 | 2 | **16** | 0 | 1 | 0 | 1 | **2** |

Each component was scored 0-3, and “cellular crescents” and “fibrinoid necrosis or karyorrhexis” was in addition weighted by a factor of 2. Mice were grouped according to this scoring; Minimal nephritis (Min): activity index ≤2, chronicity index 0. Moderate nephritis (Mod): activity index >2, chronicity index 0. Severe nephritis (Sev): chronicity index ≥1.
