# Supplementary figures and images for: Lupus nephritis progression in FcγRIIB-/-yaa mice is associated with early development of glomerular electron dense deposits and loss of renal DNase I in severe disease
Source: PLoS One. 2017 Nov 30;12(11):e0188863. doi: 10.1371/journal.pone.0188863 (PMC5708736; doi:10.1371/journal.pone.0188863)

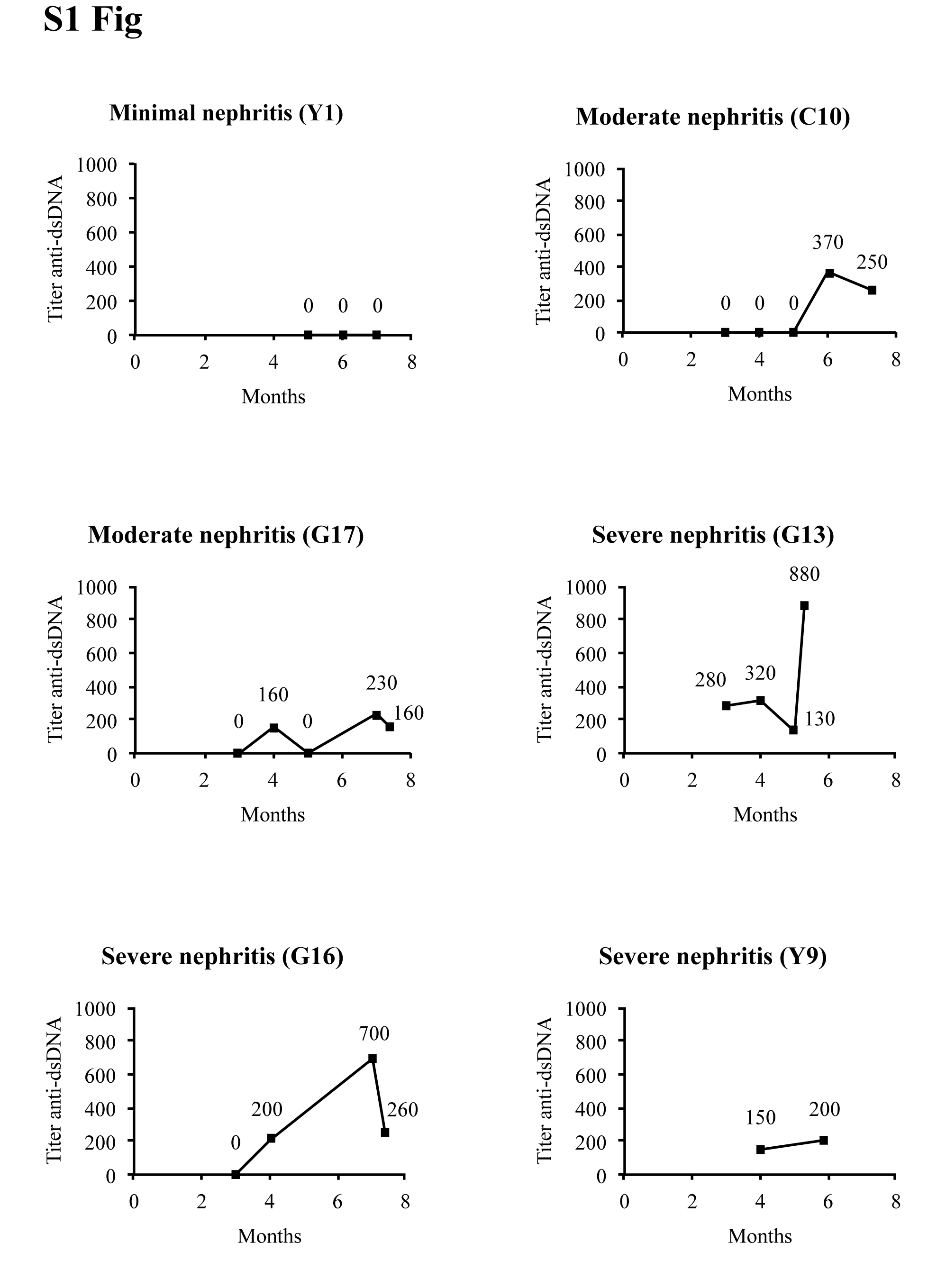

Supplement: S1 Fig — 6/25 mice had sera available for anti-dsDNA ELISA at several time points in addition to end stage sera. (TIF) [file pone.0188863.s002.tif]
